# Electrochemical Detection of Levofloxacin Using a Polydopamine-Based Molecular Imprinting Polymer

**DOI:** 10.3390/molecules31010052

**Published:** 2025-12-23

**Authors:** Alessandro Lo Presti, Fabricio Nicolas Molinari, Chiara Abate, Enza Fazio, Carmelo Corsaro, Ottavia Giuffrè, Anna Piperno, Giulia Neri, Claudia Foti

**Affiliations:** 1 Department of Chemical, Biological, Pharmaceutical, and Environmental Sciences, University of Messina, 31 Viale F. Stagno d’Alcontres, 98166 Messina, Italy; alessandro.lopresti@studenti.unime.it (A.L.P.); fmolinari@unime.it (F.N.M.); ottavia.giuffre@unime.it (O.G.); anna.piperno@unime.it (A.P.); cfoti@unime.it (C.F.); 2 Department of Mathematical and Computer Sciences, Physical Science and Earth Science, University of Messina, 31 Viale F. Stagno d’Alcontres, 98166 Messina, Italy; enza.fazio@unime.it (E.F.); carmelo.corsaro@unime.it (C.C.)

**Keywords:** molecularly imprinted polymer, dopamine, levofloxacin, screen-printed carbon electrodes, electrochemical sensor

## Abstract

The integration of molecular imprinting technology with electrochemical methods has become fundamental in the development of next-generation sensors. This study explores two different strategies for developing a dopamine-based molecularly imprinted polymer (MIP) for the electrochemical sensing of levofloxacin. In the first case, the MIP is developed by electropolymerization on a screen-printed carbon electrode (SPCE) surface using cyclic voltammetry, while in the second, the MIP is obtained by an oxidation process, and the resulting dispersion is drop-cast on the SPCE surface. The same approach is used for a non-imprinted polymer. The physicochemical properties of the synthesized materials and the surface morphology of the modified electrodes are investigated by several techniques. Differential pulse voltammetry is used to evaluate the performance of the modified electrodes, assessing their linear concentration range, limit of detection, and limit of quantification, together with repeatability and selectivity. MIP-based SPCEs obtained with these two fabrication strategies exhibited comparable imprinting factor values and linear concentration ranges, along with comparable limits of detection and quantification. The MIP-based SPCE obtained by electropolymerization showed greater repeatability, whereas the MIP-based SPCE produced by drop-casting provided higher sensitivity in levofloxacin detection.

## 1. Introduction

Levofloxacin (LEV) is a fluoroquinolone (FQ) antibiotic with good bactericidal activity against a broad spectrum of Gram-positive and Gram-negative bacteria [1,2,3]. It is also used to treat skin and kidney infections, as well as urinary and chronic respiratory diseases [2,3]. Although it possesses proven therapeutic efficacy and widespread approval, the significance of LEV misuse, such as its effects on food safety, environmental contamination, and antimicrobial resistance, cannot be ignored [1]. Its environmental accumulation, especially in water, is a critical issue due to the related ecological dangers and safety risks, as well as the emergence of drug-resistant bacteria [4,5]. Therefore, it is of great importance to monitor LEV levels in the aquatic environment using highly sensitive methodologies.

To this end, electrochemical techniques offer a convenient, versatile, and rapid alternative to conventional detection methods, including capillary electrophoresis, chemiluminescence, enzyme-linked immunosorbent assays (ELISAs), and high-performance liquid chromatography (HPLC) [1,2,4,6]. In particular, for LEV electrochemical detection, the employment of suitable modified electrodes leads to an enhancement in sensor performance [2,4].

Recently, molecularly imprinted polymers (MIPs) [7], engineered with cavities tailor-made for a specific target molecule, have attracted special attention as chemical modifiers of the electrode working area [8]. This approach improves the analytical sensitivity and selectivity of the sensors, providing a wide linear concentration range and a low limit of detection (LOD) [9,10,11]. Moreover, their easy preparation route, as well as their speed, cost-effectiveness, and reproducibility, allow their employment in the detection of several molecules, including antibiotics [1,6,8,9,12,13,14,15,16,17,18,19,20,21,22,23,24].

Polydopamine (polyDA) is one of the most effective organic coating materials owing to its strong adhesion to a wide variety of substrates, even under aqueous conditions, without the need for additional linkers [25,26]. PolyDA is inexpensive and easy to prepare through the oxidation of the dopamine (DA) monomer. Consequently, polyDA has been employed to prepare various sensors, including MIP-based sensors and biosensors, with promising results [27,28,29,30,31].

Furthermore, polyDA showed a great affinity toward LEV due to the possibility of establishing various electrostatic interactions and hydrogen bonds between the catechol, quinone and amine groups of polyDA, and the carboxyl, ketone, piperazinyl and fluorine groups of LEV [27,28,29]. In this study, polyDA-modified MIP electrodes for the detection of LEV were prepared using two different strategies: electropolymerization and drop-casting. The former is a simple and rapid procedure that offers high scalability, accuracy, selectivity and sensitivity [10,32,33]. However, the electropolymerized film can exhibit poor conductivity, resulting in scarce sensitivity and a narrower detection range [1,34]. On the other hand, the drop-casting procedure is equally simple in preparation; however, the difficulty in achieving a homogenous coating of the electrode surface and the lack of control over thickness must be taken into account [35].

**Scheme 1:**
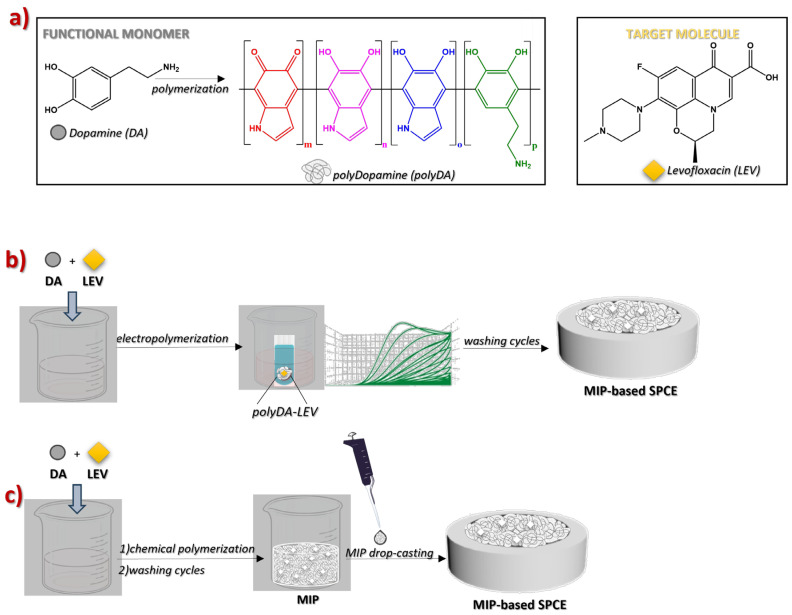
(**a**) Chemical structures of polymerized dopamine (polyDA) and levofloxacin (LEV); (**b**) schematic representation of preparation of MIP-based SPCE by electropolymerization using cyclic voltammetry (CV, voltammograms are reported in green); and (**c**) drop-casting approach.

In this study, a MIP-based approach was adopted for LEV sensing following two methodologies (Scheme 1), i.e., (i) electropolymerization and (ii) material synthesis and subsequent drop-casting of the dispersion on the surface of SPCEs. Under the same experimental conditions, non-imprinted polymers (NIPs) were prepared. The chemical composition of MIP and NIP was studied by thermogravimetric analysis (TGA) and attenuated total reflection–Fourier transform infrared (ATR-FTIR) measurements, while colloidal stability was explored by dynamic light scattering (DLS) analysis. The functionalization of the electrode surfaces was monitored by cyclic voltammetry (CV) and differential pulse voltammetry (DPV) using K_3_Fe(CN)_6_ as a redox probe, and further confirmed by X-ray photoelectron spectroscopy (XPS) measurements and microscope images. The formation of imprinted cavities was evaluated by calculating the imprinting factor (IF) [5,36]. The linear concentration range was 0.066 ≤ [LEV]/μmol L^−1^ ≤ 0.26 for both MIP-based SPCEs. While the drop-casting method provides the lowest LOD values and limit of quantification (LOQ) and good results in terms of IF and sensitivity, electropolymerization led to advantageous repeatability (3.5%). 

## 2. Results and Discussion

### 2.1. MIP/NIP-Based Sensor Preparation

MIP/NIP-based SPCEs were prepared using two different strategies: (i) electropolymerization and (ii) drop-casting, respectively.

#### 2.1.1. Electropolymerization

**Figure 1:**
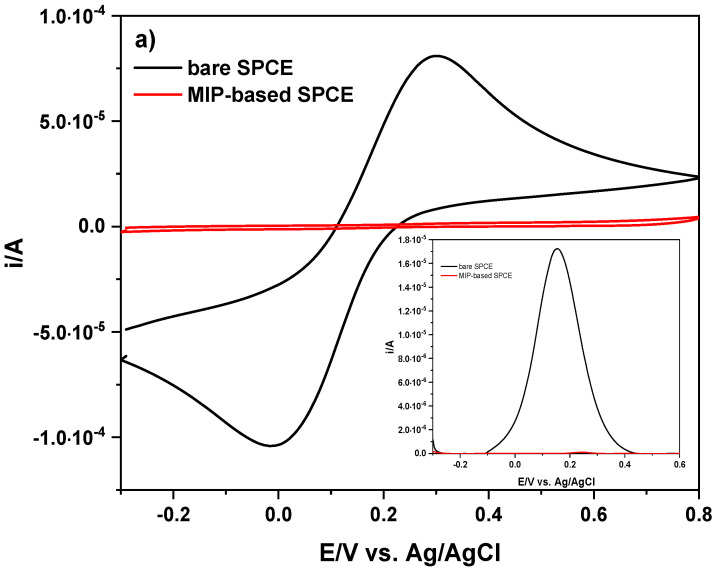

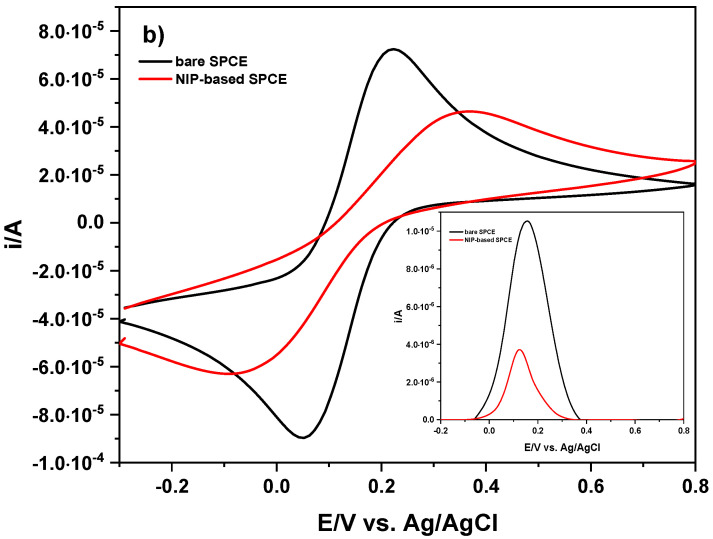
(**a**) CV and DPV scans (in the inset) of K_3_Fe(CN)_6_ (5 mmol L^−1^) in KCl (0.1 mol L^−1^) on bare SPCE (black line) and SPCE modified with MIP (red line), obtained through electropolymerization. (**b**) CV and DPV scans of K_3_Fe(CN)_6_ (5 mmol L^−1^) in KCl (0.1 mol L^−1^) on bare SPCE (black line) and SPCE modified with NIP (red line), obtained through electropolymerization.

Electropolymerization was performed in situ by CV, carrying out 40 scans from −0.2 V to +0.8 V (vs. Ag/AgCl) at 20 mV s^−1^, using phosphate buffer (PB, pH 7) solutions containing DA/LEV (10:8 mmol L^−1^) or DA (10 mmol L^−1^) for MIP or NIP preparation, respectively. Further details on the electropolymerization conditions are reported in the experimental section (Section 3.5). The progression of both polymerization processes is shown in Figure S1, where a decrease in peak current is evident as the number of cycles increases. In the first scan of Figure S1, an anodic oxidation signal clearly occurs on both electrode materials at about 380 mV (vs. Ag/AgCl). This signal, whose initial anodic peak current (i_ap_) is ~140.0 μA, decreases after the second scan and progressively until decay, indicating the depletion of the monomer at the electrode surface and formation of insulating film. The voltammetric response of all SPCEs were monitored by CV and DPV of K_3_Fe(CN)_6_, voltammograms of which are depicted in Figure 1. In particular, Figure 1a shows that the two well-defined redox peaks in the unaltered SPCE undergo complete attenuation in the peak currents (i/A). This decrease in i/A is indicative of the formation of a non-conductive polymeric film on the electrode surface that, in turn, hinders the reactive exposure to the electrode surface in the MIP- and NIP-based SPCEs and, thus, electron transfer. A similar behavior is shown in the inset of Figure 1a related to the anodic peak current (i_ap_/A) and, thus, to the [Fe(CN)_6_]^4−/3−^ species. The decrease in terms of the i_pa_/A values is much more pronounced for the MIP-based SPCE (inset of Figure 1a) than the NIP-based SPCE (Figure 1b).

**Figure 2:**
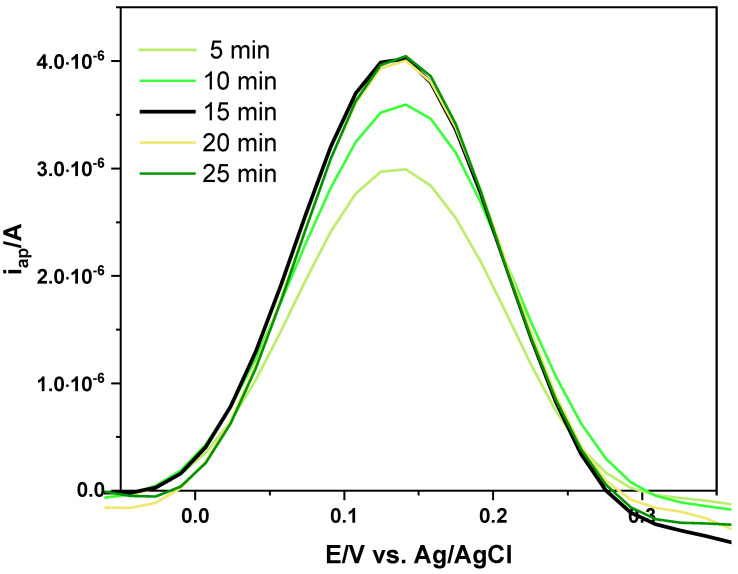
DPV scans of [Fe(CN)_6_]^4−/3−^ (5 mmol L^−1^) in KCl (0.1 mol L^−1^) on MIP-based SPCE after a washing period ranging from 5 (light green) to 25 min (olive green) in H_2_SO_4_ (0.1 mol L^−1^). The black voltammogram of [Fe(CN)_6_]^4−/3−^ corresponds to a washing time of 15 min in H_2_SO_4_ (0.1 mol L^−1^).

To activate the molecular recognition sites by removing the template molecule (LEV), the washing solvent and removal time must be carefully chosen. Figure 2 and Figure S2 show the voltammograms after washing the MIP-based SPCEs with different solvents (i.e., H_2_SO_4_, deionized water, H_2_O/MeOH 1:1 *v*/*v*, and CH_3_COOH/MeOH 1:9 *v*/*v*). In water, the electrochemical signal did not increase and, thus, water was unable to remove LEV. In other solvents (H_2_O/MeOH 1:1 *v*/*v*, CH_3_COOH/MeOH 1:9), the signals increased up to 20 min, but the electrochemical response after the washing phase was not linearly dependent on LEV concentration. H_2_SO_4_ (0.1 mol L^−1^) was therefore chosen as the solvent considering the increase in signal with the washing time and the subsequent voltammetric response of the electrode. Indeed, Figure 2 highlights a gradual increase in the i_ap_ values of the redox probe up to 15 min (black voltammogram in Figure 2) and subsequent stabilization of its electrochemical signal, showing that imprinted cavities in MIP films successfully formed. A washing time of 15 min was considered sufficient to achieve greater accessibility of the electrode surface, due to the creation of cavities. Therefore, this washing step was applied after both the electropolymerization and each DPV measurement in the presence of LEV.

Imprinting performance was evaluated by calculating the imprinting factor (IF), defined as the ratio between the i_ap_/A values of MIP- and NIP-based SPCEs in the presence of LEV (0.1 mmol L^−1^, Figure S3). The IF value of 1.9 confirms the successful generation of cavities within the MIP matrix [5].

**Figure 3:**
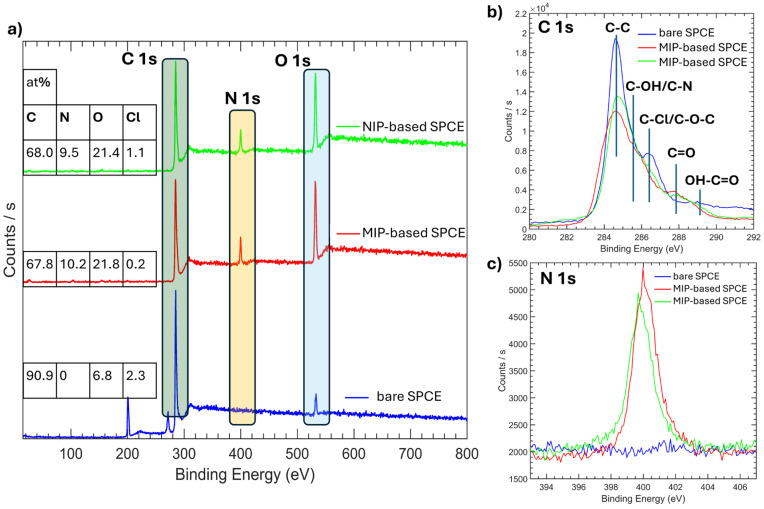
(**a**) XPS results showing survey spectra and elemental composition, as well as high-resolution (**b**) C 1s and (**c**) N 1s profiles of the bare SPCE and the MIP- and NIP-based SPCEs.

Further confirmation of the functionalization of SPCEs was obtained by XPS measurements (Figure 3). As expected, MIP- and NIP-based SPCEs exhibited a different surface composition with respect to the bare SPCE in terms of C (from 68 to 91%), O (from 21% to 7%), and N (from 10% to 0%) content. Chlorine is mainly present only in the bare electrode, probably due to the cleaning procedure performed during production; its disappearance after electrode functionalization confirms good electrode coverage. The C 1s profile of all samples showed a main contribution at 284.5 eV due to C−C bonds, along with additional contributions at higher binding energies attributed to carbon atoms bonded to nitrogen or oxygen in different chemical configurations (Figure 3b), more evident for the MIP- and NIP-based SPCEs. Notably, we see the appearance of nitrogen contribution when the electrode surfaces are functionalized with MIP and NIP (Figure 3c), further providing coverage of electrode surfaces with polyDA.

#### 2.1.2. Synthesis of MIP and Drop-Casting Procedure

MIP was prepared by polymerizing DA in the presence of the template, LEV. Specifically, polydopamine (polyDA) was obtained by an oxidation process performed in phosphate buffer (PB) solution in the presence of piperidine. To ensure effective removal of LEV, the supernatant solutions collected after each washing cycle were analyzed by UV-vis spectroscopy. The optimal number of washing cycles was set at ten, as the two typical absorption bands (286, 331 nm) of LEV (Figures S4 and S5) were no longer detectable. The same synthetic procedure was adopted for NIP, omitting LEV.

**Figure 4:**
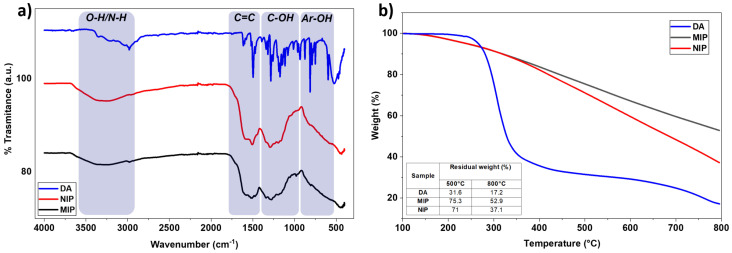
(**a**) ATR-FTIR spectra of DA (blue line), MIP (black line) and NIP (red line) (main contributions are highlighted in light blue). (**b**) TGA profiles of DA (blue line), MIP (black line) and NIP (red line) recorded in nitrogen atmosphere.

The ATR-FTIR spectra of MIP and NIP were very similar, showing broad bands with less defined peaks than those of DA, due to their more complex polymeric structure (Figure 4a). In particular, the band between 3675 and 3000 cm^−1^ is related to the O-H and N-H vibrational stretching modes, while the two broad bands between 1807 cm^−1^ and 915 cm^−1^ are attributed to the C=C vibrational stretching of the aromatic ring and the bending vibration of the C-OH and Ar-OH bonds [37,38].

TGA analyses indicate that neither MIP nor NIP display a distinct decomposition temperature, unlike DA, confirming the structural heterogeneous nature of polyDA (Figure 4b), in accordance with the literature [39]. At 800 °C, the residual masses of MIP and NIP were 52.9% and 37.1%, respectively, values significantly higher than those of DA (17.2%) and correlated with a carbonization process occurring in polyDA.

The colloidal stability of MIP and NIP was investigated by DLS analysis (Figure S6). The MIP dispersion exhibited an average hydrodynamic diameter of 309 ± 7 nm, slightly higher than that of NIP (297 ± 26 nm). Moreover, both MIP ad NIP dispersions showed good polydispersity indexes (PDI) of 0.39 and 0.47, respectively. Values of zeta potential (ZP) were measured for MIP and NIP, and were −38.8 ± 1.2 and −37.1 ± 0.7 mV, respectively, in line with the high abundance of oxygenated functional groups.

Next, 1 μL of PB solution containing MIP or NIP was cast five times on the electrode surfaces, and the modified SPCEs were dried under vacuum (40 °C).

To investigate the differences in surface morphology among the modified electrodes, high-resolution 3D digital microscopy at a micrometric scale was used.

**Figure 5:**
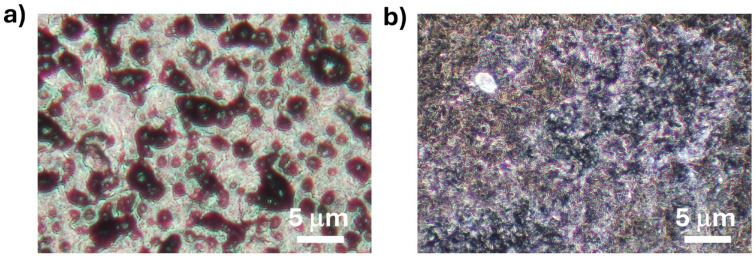
Comparison of the surface morphology of MIP-based SPCEs prepared by (**a**) drop-casting and (**b**) electropolymerization. The images were collected by using a Hirox 3D digital microscope.

No significant morphological differences between the MIP and NIP samples were observed (see Figure S7). In the case of the SPCEs modified by the drop-casting approach, both MIP (Figure 5a) and NIP samples appear as black agglomerates, measuring a few micrometers, resulting from uneven coverage of the electrode surface. This effect may be attributed to the vacuum-drying phase. Conversely, the electrode surface coated by electropolymerization showed a uniform distribution of the MIP sample (Figure 5b), even on a smaller scale than the previous one.

These experimental results clearly highlight that the two preparation strategies lead to completely different electrode surface coverage.

**Figure 6:**
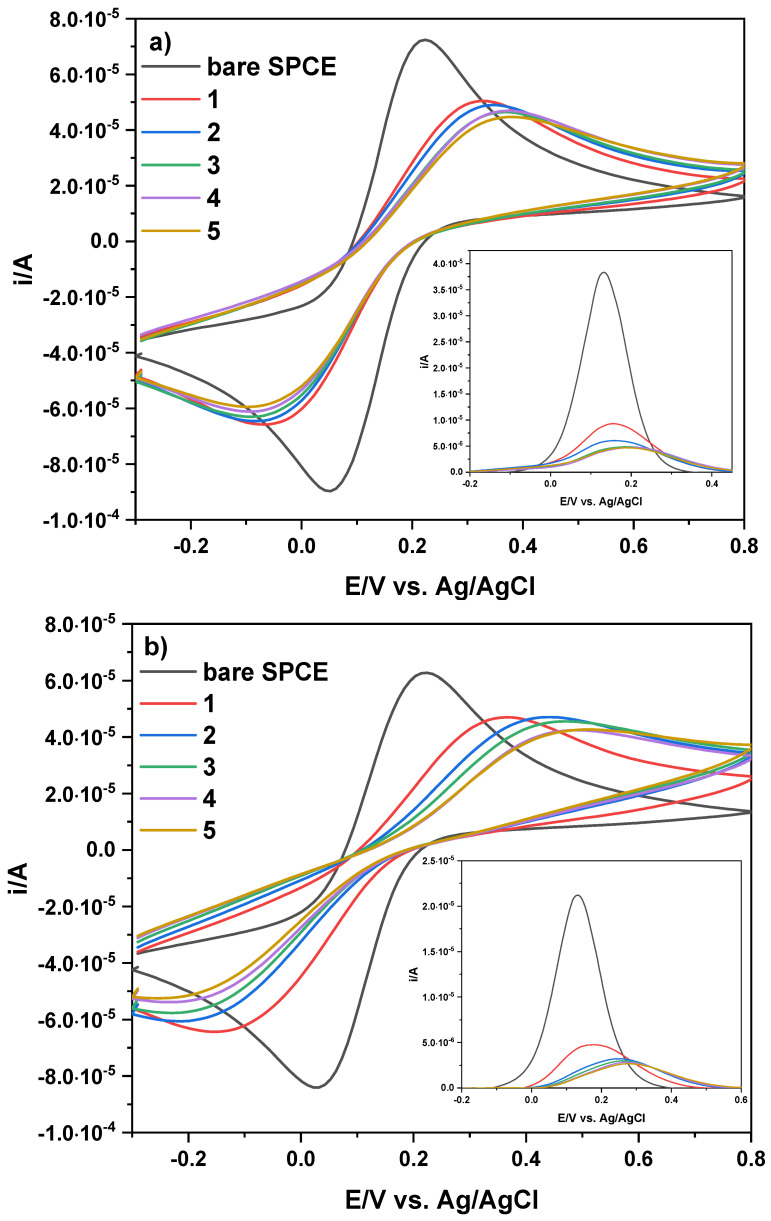
(**a**) CV and DPV scans (in the inset) of K_3_Fe(CN)_6_ (5 mmol L^−1^) in KCl (0.1 mol L^−1^) on the bare SPCE (gray line) and SPCE modified with 5 drops (each of 1 μL, see from the red line to the ochre line) of a PB solution containing MIP (2 mg mL^−1^), which was previously synthetized. (**b**) CV and DPV scans (in the inset) of K_3_Fe(CN)_6_ (5 mmol L^−1^) in KCl (0.1 mol L^−1^) on the bare SPCE (gray line) and SPCE modified with 5 drops (each of 1 μL, see from the red line to the ochre line) of a PB solution containing NIP (2 mg mL^−1^), which was previously synthetized.

The voltammetric response of the electrodes modified by the drop-casting approach was evaluated by CV and DPV using K_3_Fe(CN)_6_ as the redox probe. Figure 6 shows CV and DPV scans obtained on the bare SPCE (gray line) and modified SPCE with 5 drops (each of 1 μL) of a PB solution containing MIP (2 mg mL^−1^), which was previously synthetized. By increasing the film layer on the electrode surface, both the anodic and cathodic signals of the redox probe sharply decrease following the first addition of MIP (~30.0%) and, to a lesser extent, after subsequent additions (~3.0% each time). In particular, the anodic and cathodic signals of K_3_Fe(CN)_6_ on the bare SPCE are E_ap_ = 221.0 mV, i_ap_ = 73.1 μA, E_cp_ = 47.0 mV, and i_cp_ = 90.3 μA, and change to E_ap_ = 375.0 mV, i_ap_ = 45.1 μA, E_cp_ = −89.0 mV, and i_cp_ = 59.6 μA after the last drop-casting of PB solution containing the synthetized MIP. Ultimately, E_ap_ values undergo a shift toward positive potentials (∆E_ap_ = 154.0 mV vs. Ag/AgCl) from the bare SPCE with respect to the modified ones, while E_cp_ values undergo a lesser effect in the opposite direction (∆E_cp_ = 42.0 mV vs. Ag/AgCl). For clarity, Table S1 summarizes the change in terms of potential and peak currents. On the other hand, the anodic peak current decreases more (∆i_ap_ = 38.3%) than the cathodic peak current (∆i_cp_ = 34.0%) when moving from the bare electrode surface (gray CV) to the last MIP layer added on the SPCE (ochre line in Figure 6). This decrease in anodic peak current (∆i_ap_ = 87.0%) is best highlighted in the inset of Figure 6, together with no significant change in the anodic signal of K_3_Fe(CN)_6_ after the third drop-casting of PB solution containing MIP, indicating saturation of the electrode surface and suggesting signal-off behavior. For the NIP-based SPCE, CV and DPV scans are shown in Figure 6b. After pouring the first drop of PB solution containing NIP onto the electrode surface, both the anodic and cathodic signals of the redox probe decrease (~24.4%). Slight variations in the signals occur with the addition of further aliquots of solution onto the electrode surface.

In particular, the anodic and cathodic signals of K_3_Fe(CN)_6_ on the bare SPCE are E_ap_ = 219.0 mV, i_ap_ = 63.4 μA, E_cp_ = 26.0 mV, and i_cp_ = 84.6 μA, and change to E_ap_ = 508.0 mV, i_ap_ = 42.9 μA, E_cp_ = −242.0 mV, and i_cp_ = 52.9 μA after the last drop-casting of PB solution containing the synthetized NIP. Therefore, E_ap_ values undergo a considerable shift toward positive potentials (∆E_ap_ = 289.0 mV vs. Ag/AgCl) from the bare SPCE to the modified SPCEs, while E_cp_ values undergo a shift in the opposite direction (∆E_cp_ = 216.0 mV vs. Ag/AgCl). Table S1 also summarizes the change in potential and peak currents related to the NIP-based SPCE. The potential changes (∆E_ap_ and ∆E_cp_) have a greater effect than those observed in the MIP-based SPCE, probably due to the absence of cavities available to the redox probe. In this case, the ∆i_ap_ and ∆i_cp_ values are 32.4% and 37.5%, respectively, when moving from the bare electrode surface (gray CV) to the last NIP layer added on the SPCE (ochre line in Figure 6b). However, to compare the change in the anodic peak of the MIP, anodic peaks for the NIP-based SPCE are again pointed out in the inset of Figure 6b). A similar result (∆i_ap_ = 87.0%) was obtained for MIP- and NIP-based SPCEs.

As performed for the electropolymerized MIP-based SPCE, the IF was evaluated as the ratio between the i_pa_ values of the MIP- and NIP-based SPCEs in the presence of LEV (0.1 mmol L^−1^, Figure S3). In this case a lower IF value (1.3) was obtained compared to the one previously computed for the electropolymerized electrode. This effect could be due to the different coatings of electrode surfaces, as shown in Figure 5.

### 2.2. Electroanalytical Sensing

**Figure 7:**
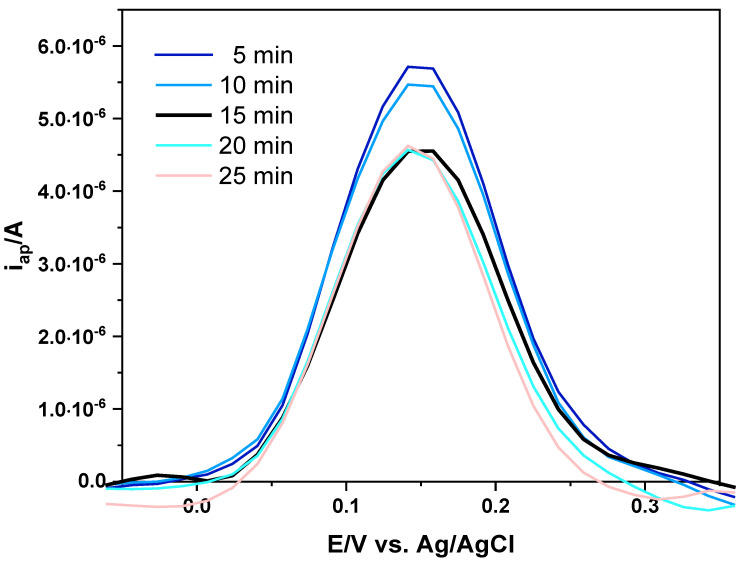
DPV scans of [Fe(CN)_6_]^4−/3−^ (5 mmol L^−1^) in KCl (0.1 mol L^−1^) on the MIP-based SPCE after an incubation period ranging from 5 (blue line) to 25 min (pink line) in LEV (0.1 mmol L^−1^). The black voltammogram of [Fe(CN)_6_]^4−/3−^ corresponds to an incubation time of 15 min in LEV (0.1 mmol L^−1^).

The MIP-based sensors consisting of a polyDA layer, obtained by using the two different procedures, were tested to detect LEV in PB solution (pH 7). Preliminarily, the time required for LEV to re-soak the cavities of the film on the electrode surface was evaluated. Incubation time is an important factor and directly affects the efficiency of molecular recognition: inadequate exposure resulted in incomplete capture of the template molecule, while prolonged incubation did not provide further improvements. MIP-based SPCEs were immersed in PB solution containing LEV (0.1 mmol L^−1^) for 5, 10, 15, 20 and 25 min, and DPV measurements of K_3_Fe(CN)_6_ were recorded (Figure 7). Unlike the washing phase (Figure 2), in which the i_ap_/A values increased over time due to enhanced accessibility, incubation resulted in a progressive decrease in i_ap_, attributable to the re-binding of LEV within the MIP cavities, thus hindering the diffusion of the redox probe ([Fe(CN)_6_]^4−/3−^). An incubation period of 15 min was sufficient to achieve binding equilibrium between LEV and the sites imprinted in the MIP film on the electrode surface (Figure 7, black line). Therefore, 15 min was established as the optimal incubation time and used in all subsequent studies.

**Figure 8:**
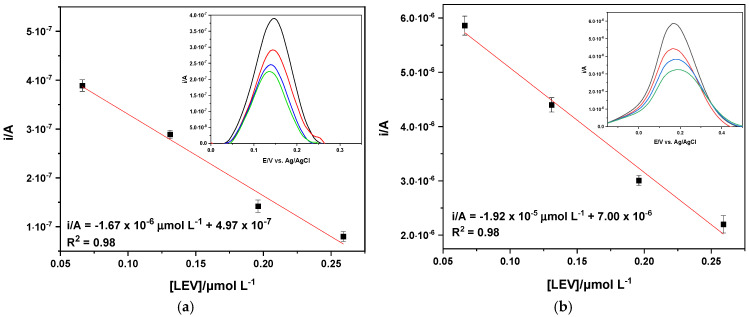
i_ap_ values vs. LEV concentration obtained by DPV scans (in the insets) of K_3_Fe(CN)_6_ (5 mmol L^−1^) in KCl (0.1 mol L^−1^) with the MIP-based SPCEs functionalized by (**a**) electropolymerization and (**b**) drop-casting.

The analytical performance of the MIP-based SPCEs was assessed via voltammetric titrations in PB solution, considering, in turn, different concentrations of LEV (total range 0.002 ≤ µmol L^−1^ ≤ 42). The electrochemical response was evaluated by DPV of K_3_Fe(CN)_6_. For both type of SPCEs, i_ap_ values linearly decreased with LEV concentration in the range (0.066 ≤ μmol L^−1^ ≤ 0.26, R^2^ = 0.98), as shown in Figure 8. Although the linear concentration ranges were the same, the sensitivity (s) (μA μLmol^−1^) of the two types of electrodes was very different, with s = 1.67 and 19.2 μA μLmol^−1^ for the electropolymerization and drop-casting methods, respectively. The limit of detection (LOD) and limit of quantification (LOQ) were determined to be 3 σs^−1^ and 10 σs^−1^ (s is the slope of the calibration curve, σ is the standard deviation of the intercept) [40]. LOD and LOQ values of 0.053 μmol L^−1^ and 0.18 μmol L^−1^, and 0.045 μmol L^−1^ and 0.15 μmol L^−1^, were obtained for electropolymerization and drop-casting, respectively.

The analytical parameters obtained by MIP-based SPCEs are given in Table 1. Independently of the experimental procedure employed, the anodic peak currents (i_ap_/A) linearly decrease as LEV concentration increases within the range (0.066 ≤ [LEV]/μmol L^−1^ ≤ 0.26). This behavior may be attributed to LEV molecules filling the cavities in the MIP film, thereby preventing the diffusion of the redox species ([Fe(CN)_6_]^4−/3−^) and leading to reduced i_ap_ values. The other analytical parameters, such as LOD (0.053 and 0.045 μmol L^−1^) and LOQ (0.18 and 0.15 μmol L^−1^), are very similar. In any case, LOD values are an order of magnitude lower with respect to the ones reported in the literature, as shown in Table S2 [1,2,6,41,42,43].

The repeatability was estimated based on the relative standard deviation (RSD%) of three DPV measurements of K_3_Fe(CN)_6_ after immersing the differently modified SPCEs in a PB solution containing LEV (0.26 μmol L^−1^) for 15 min. In addition, each measurement was interspaced with H_2_SO_4_ (0.1 mmol L^−1^) washes. The voltammograms are shown in Figure S8. Values of 3.5% and 4.8% were obtained for MIP-based SPCEs obtained through electropolymerization and drop-casting, respectively.

**Figure 9:**
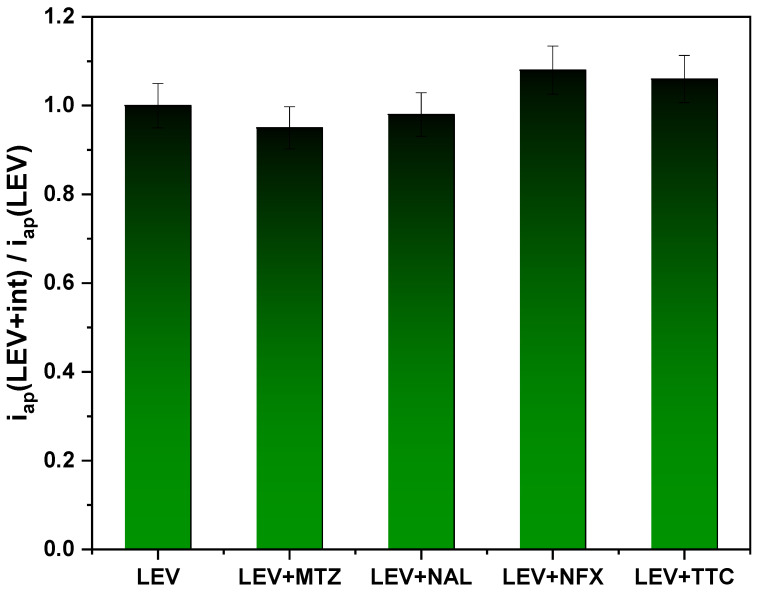
Ratio of the i_ap_ values in PB solutions containing LEV in presence of potential interfering species (i_ap(LEV+interf)_) in a 1:1 molar ratio, and in only LEV 0.1 μmol L^−1^ (i_ap(LEV)_).

Finally, selectivity was evaluated considering the ability of the sensor to also measure the target molecule in the presence of a potentially interfering water-soluble antibiotic, such as metronidazole (MTZ), nalidixic acid (NAL), norfloxacin (NFX) or tetracycline (TTC). DPV scans for the redox probe were performed after dipping the MIP-based SPCE in PB solutions containing only LEV (0.1 μmol L^−1^) or LEV with interfering species (in a 1:1 μmol L^−1^ ratio), for 15 min. The effect of the presence of interfering species was evaluated considering the ratio between the i_ap_ values in the presence (i_ap(LEV+int)_) and in the absence of interfering species (i_ap(LEV)_). Values very close to the unit value (Figure 9) indicate a negligible effect of the presence of potentially interfering antibiotic [44].

## 3. Materials and Methods

### 3.1. Materials

Dopamine hydrochloride (DA), levofloxacin (LEV, 98.0%), potassium ferricyanide(III) (K_3_Fe(CN)_6_, ≥99.0%), potassium chloride (KCl, extra pure) and sodium chloride (NaCl, ≥99.0%) were purchased from Sigma-Aldrich/Merck, Darmstadt, Germany. NaCl was dried at 110.0 °C before its use and dissolved in deionized water; phosphate buffer (PB, pH 7.0) was prepared by weighing and dissolving the respective salts (KCl, KH_2_PO_4_ and Na_2_HPO_4_ H_2_O; both phosphate salts were purchased from Fluka/Merck, Darmstadt, Germany, puriss. p.a. ≥ 99.0%). K_3_Fe(CN)_6_ solutions (5 mmol L^−1^) were prepared in KCl (0.1 mol L^−1^). Methanol (MeOH, Sigma-Aldrich/Merck KGaA, Darmstadt, Germany) and acetic acid (CH_3_COOH, Carlo Erba, Cornaredo, Italy) were used for the removal template, namely in the washing steps. Solutions of sulfuric acid (H_2_SO_4_, 96.0%, Sigma-Aldrich), hydrochloric acid (HCl 37%, Sigma-Aldrich/Merck KGaA, Darmstadt, Germany) and sodium hydroxide (NaOH 32.0%, Fluka, Pistoia, Italy) were prepared by diluting the respective products. HCl and NaOH were standardized with sodium carbonate and potassium acid phthalate, both acquired from Sigma-Aldrich (≥99.5%) and pre-dried at 110.0 °C. For the selectivity study, the following antibiotics were used: nalidixic acid sodium salt (NAL ≥ 99.0%, Sigma-Aldrich/Merck KGaA, Darmstadt, Germany), tetracycline hydrochloride (TTC 96.0%, Alfa Aesar/Fisher Scientific, Milan, Italy), norfloxacin (NFX 98.0%, Sigma-Aldrich/Merck KGaA, Darmstadt, Germany) and metronidazole (MTZ 99.0%, Alfa Aesar/Fisher Scientific, Milan, Italy).

### 3.2. Synthesis of MIP/NIP-Based SPCE by Drop-Casting

DA (176.7 mg, 0.932 mmol) and LEV (260 mg, 0.72 mmol) were dissolved in 90 mL of PB (pH 7), and the reaction was incubated for 30 min in an ice bath under magnetic stirring. Then, piperidine (120 µL, 1.2 mmol) was added, and the reaction mixture was kept at 40 °C under magnetic agitation for 5 h. After polymerization, MIP was centrifuged (14,500 rpm, 10 min) and washed with MqW by alternating sonication and centrifugation steps. After each washing cycle, the presence of DA and LEV in the supernatant was analyzed by UV-Vis spectroscopy until no presence of LEV was observed (Figures S4 and S5). The MIP was then freeze-dried, yielding 7.1 mg of product.

Non-imprinted polymer (NIP) was synthetized following the same procedure in absence of LEV, yielding 7.4 mg of product.

The MIP-based SPCE or NIP-based SPCE was prepared by drop-casting 1 μL of the PB solution containing MIP/NIP (2 mg mL^−1^), followed by drying under vacuum at 40 °C. This procedure was repeated five times.

### 3.3. Physicochemical Characterization

ATR-FTIR spectra were registered on a Spectrum Two Perkin Elmer (Waltham, MA, USA) spectrometer equipped with a Universal Attenuated Total Reflectance Accessory (UATR).

DLS measurements (size and z-potential) were performed on a Malvern Zetasizer pro—blue label instrument (Malvern, UK). Measurements were performed at 25° C. Approximately 1 mL of sample solution was transferred into the cylindrical scattering cell.

UV-vis spectra were recorded on a Jasco V-730 spectrophotometer (Jasco, Easton, MD, USA) using quartz cells with 1 and 0.5 cm path lengths.

TGA was performed using a Discovery SDT650 (TA Instruments, New Castle, DE, USA). The test method involved isothermal at 100 °C for 20 min to eliminate humidity followed by a ramp of 10 °C min^−1^ until 800 °C.

X-ray photoelectron spectroscopy (XPS) was performed under ultrahigh vacuum (UHV) conditions using a UK Thermo Scientific (Rodano, MI, Italy) K-Alpha spectrometer equipped with a monochromatic Al Kα source (hν = 1486.6 eV) and a hemispherical analyzer. The constant-pass energy was set at 200 eV for the survey scans and 50 eV for the high-resolution XPS spectra.

Optical images were acquired by using a HIROX 3D digital microscopy system provided by the company Simitecno s.r.l. The microscope is equipped with a motorized rotating optics angled at 360°, which allows the actual observation angle to be changed thanks to the motorized rotation of the optics around the point of interest; a 5.0 MP CMOS sensor; a motorized stage; and different objectives with magnification up to 10,000×.

### 3.4. Voltammetric Equipment and Procedures

Electrochemical measurements were taken with a μAutolab potentiostat–galvanostat system (type III, EcoChemie, Herisau, Switzerland) interfaced with a PC-controlled electrochemical workstation with a three-electrode cell configuration (Metrohm DropSens screen-printed electrodes (SPEs), DRPDSC4MM 72098, Herisau, Switzerland). Screen-printed carbon electrodes (SPCEs, ref. C11L, Herisau, Switzerland) were used throughout, consisting of carbon working electrodes and counter-electrodes (WE and CE) and a Ag/AgCl reference electrode (RE). Voltammograms were processed by General Purpose Electrochemical System software (GPES 4.9, Eco Chemie B.V., Utrecht, The Netherlands).

### 3.5. Synthesis of MIP/NIP-Based SPCE by Electropolymerization

To prepare the MIP-based SPCE, an aliquot of PB solution containing DA (10 mmol L^−1^) and LEV (8 mmol L^−1^) was put on the electrode surface, and 40 cyclic voltammetry (CV) scans were performed from −0.2 V to +0.8 V (vs. Ag/AgCl) with a potential step and scan rate of 10 mV and 20 mVs^−1^. The washing phase was tested using several solvents (i.e., H_2_SO_4_, deionized water, H_2_O/MeOH 1:1 *v*/*v*, CH_3_COOH/MeOH 1:9 *v*/*v*) at different times (5, 10, 15, 20 and 25 min). The best result was found using H_2_SO_4_ (0.1 mol L^−1^) at 15 min of incubation.

The NIP-based SPCE was prepared following the same procedure, but in the absence of LEV.

### 3.6. Electrochemical Response Assessment of MIP/NIP-Based SPCE Prepared by Drop-Casting

The electrochemical response of the SPCEs modified by the drop-casting approach was evaluated by CV and differential pulse voltammetry (DPV), using as a redox probe a solution of K_3_Fe(CN)_6_ (5 mmol L^−1^) in KCl (0.1 mol L^−1^). Both CV and DPV were performed from −0.3 V to +0.8 V (vs. Ag/AgCl), with a step potential of 5 mV, modulation amplitude of 25 mV and scan rate of 100 mV s^−1^.

## 4. Conclusions

This study aimed to develop an analytical easy-to-use method for designing electrochemical sensors. To this end, MIP- and NIP-based SPCEs were fabricated adopting two functionalization strategies, (i) electropolymerization and (ii) drop-casting. The systems were fully characterized, and their electrochemical performance was compared. The main differences among the analytical performance of the two MIP based sensors are the sensitivity and repeatability. Although both approaches showed a similar downward trend, the drop-casting method resulted in improved sensing performance (s = 19.2 μA μLmol^−1^ against 1.67 μA μLmol^−1^ when using electropolymerization). The 91.3% increase in sensitivity observed for drop-casting during LEV detection can be attributed to higher electrical conductance and reduced aggregation of MIP compared to the polymeric film on the SPCE modified via electropolymerization. The slightly lower imprinting factor (IF 1.3) value obtained for the drop-cast sensor could be due to the lack of uniform coverage of the material on the electrode surface after the vacuum-drying step.

On the other hand, the electropolymerized MIP sensor exhibited superior IF (1.9) and repeatability (3.5%) compared to the drop-casting system (4.8%), confirming its potential as a reliable and environmentally friendly strategy for directly depositing the MIP layer onto the SPCE surface. Moreover, electropolymerization offers advantages such as reduced time, reagent and solvent consumption, as well as fast reaction and analyses. The electrochemical sensor produced through this method enables direct (in situ) signal acquisition, providing real-time information for process monitoring.

Future work will focus on optimizing the method and evaluating its applicability to real systems, such as in wastewater and biological samples.

## Figures and Tables

**Table 1:** Analytical parameters obtained for the two MIP-based SPCEs.

	Electropolymerization	Drop-Casting
Linear range (μmol L^−1^)	0.07–0.26	0.07–0.26
Sensitivity (μA μLmol^−1^)	1.7	19.2
LOD (μmol L^−1^)	0.053	0.045
LOQ (μmol L^−1^)	0.18	0.15
Imprinting factor (IF)	1.9	1.3
Repeatability (RSD%)	3.5	4.8

## Data Availability

The original contributions presented in this study are included in the article/Supplementary Materials; further inquiries can be directed to the corresponding authors.

## Supplementary Materials

The following supporting information can be downloaded at: https://www.mdpi.com/article/10.3390/molecules31010052/s1, Figure S1: Forty cyclic voltammetry (CV) scans for inducing electropolymerization of (a) DA/LEV (10: 8 mmol L^−1^) and (b) DA (10 mmol L^−1^) on SPCE surface; Figure S2: DPV scans of [Fe(CN)_6_]^4−/3−^ (5 mmol L^−1^) in KCl (0.1 mol L^−1^) on MIP-based SPCE after a washing period between 5 and 25 min in (a) deionized water, (b) H_2_O/MeOH 1:1 *v*/*v* and (c) CH_3_COOH/MeOH 1:9 *v*/*v*); Figure S3: DPV scans of [Fe(CN)_6_]^4−/3−^ (5 mmol L^−1^) in KCl (0.1 mol L^−1^) on MIP- (red line) and NIP-based SPCE (black line) obtained through (a) electropolymerization and (b) drop-casting after a period of incubation (15 min) in PB solution containing LEV (0.1 mmol L^−1^); Figure S4: UV-vis spectra of LEV (a) and DA (b) recorded in MqW; Figure S5: UV-vis spectra of the supernatant after 2, 4, 6, 8 and 10 washing cycles for MIP (a) and after 1, 2 and 4 washing cycles for NIP (b); Figure S6: The distribution size of both MIP (a) and NIP (b) was obtained by DLS measurements, performed at the same sample concentration of 5 µg mL^−1^; Figure S7: Comparison of the surface morphology of MIP-based SPCEs prepared by (a) drop-casting and (b) electropolymerization approaches. The images were collected by using a Hirox 3D digital microscope. Figure S8: Three DPV scans of [Fe(CN)_6_]^4−/3−^ (5 mmol L^−1^) in KCl (0.1 mol L^−1^) on the MIP-based SPCE, obtained by drop-casting, after a period of incubation (15 min) in a PB solution containing LEV (0.26 μmol L^−1^). The inset shows three DPV scans of [Fe(CN)_6_]^4−/3−^ (5 mmol L^−1^) in KCl (0.1 mol L^−1^) on the MIP-based SPCE, obtained by electropolymerization, after a period of incubation (15 min) in a PB solution containing LEV (0.26 μmol L^−1^). Each measurement was interspersed with H_2_SO_4_ (0.1 mol L^−1^) washes; Table S1: Potential values and peak currents for K_3_Fe(CN)_6_ (5 mmol L^−1^) in KCl (0.1mol L^−1^) obtained on the bare SPCE and SPCEs obtained by drop-casting of 5 drops (each of 1 μL) of PB solutions containing MIP and NIP (2 mg mL^−1^), which were previously synthetized. Table S2: Linear concentration range and LOD values reported in the literature for LEV, obtained through MIP-based electrochemical sensing, over the last 12 years.

## Abbreviations

The following abbreviations are used in this manuscript:

DA	Dopamine
polyDA	Polydopamine
LEV	Levofloxacin
FQ	Fluoroquinolone
ELISA	Enzyme-linked immunosorbent assay
HPLC	High-performance liquid chromatography
MIP	Molecularly imprinted polymer
NIP	Non-imprinted polymer
LOD	Limit of detection
LOQ	Limit of quantification
TGA	Thermogravimetric analysis
ATR-FTIR	Attenuated total reflection–Fourier transform infrared
DLS	Dynamic light scattering
XPS	X-ray photoelectron spectroscopy
PB	Phosphate buffer
NAL	Nalidixic acid sodium salt
TTC	Tetracycline hydrochloride
NFX	Norfloxacin
MTZ	Metronidazole
SPEs	Screen-printed electrodes
SPCEs	Screen-printed carbon electrodes
GPES	General Purpose Electrochemical System
WE	Working electrode
CE	Counter-electrode
RE	Reference electrode
CV	Cyclic voltammetry
DPV	Differential pulse voltammetry
PDI	Polydispersity index
ZP	Zeta potential
MP	MegaPixel
CMOS	Complementary Metal–Oxide–Semiconductor
