# Supplementary material for: Electrochemical Detection of Levofloxacin Using a Polydopamine-Based Molecular Imprinting Polymer
Source: Molecules. 2025 Dec 23;31(1):52. doi: 10.3390/molecules31010052 (PMC12787073; doi:10.3390/molecules31010052)
Supplement: Supplementary file 1 [file molecules-31-00052-s001.zip › molecules-4038823-supplementary.pdf]

Supplementary Materials

Fine-tuning of an electrochemical procedure for detection of levofloxacin based on molecularly imprinted polymers (MIPs)

Alessandro Lo Presti1§, Fabricio Nicolas Molinari1§, Chiara Abate[1],* Enza Fazio[2], Carmelo Corsaro[2], Ottavia Giuffrè[1], Anna Piperno[1], Giulia Neri[1],* and Claudia Foti

[1]Department of Chemical, Biological, Pharmaceutical, and Environmental Sciences, University of Mes-sina, 31 Viale F. Stagno d'Alcontres, 98166 Messina, Italy.

[2]Department of Mathematical and Computer Sciences, Physics Science and Earth Science, University of Messina, 31 Viale F. Stagno d'Alcontres, 98166 Messina, Italy

*Correspondence: cabate@unime.it (C.A.); giulia.neri@unime.it (G.N.)

§these authors have equally contributed

SUMMARY

Figure S1 2

Figure S2 3

Figure S3 4

Figure S4 5

Figure S5 5

Figure S6 6

Figure S7 7

Figure S8 7

Table S1 8

Table S2 9

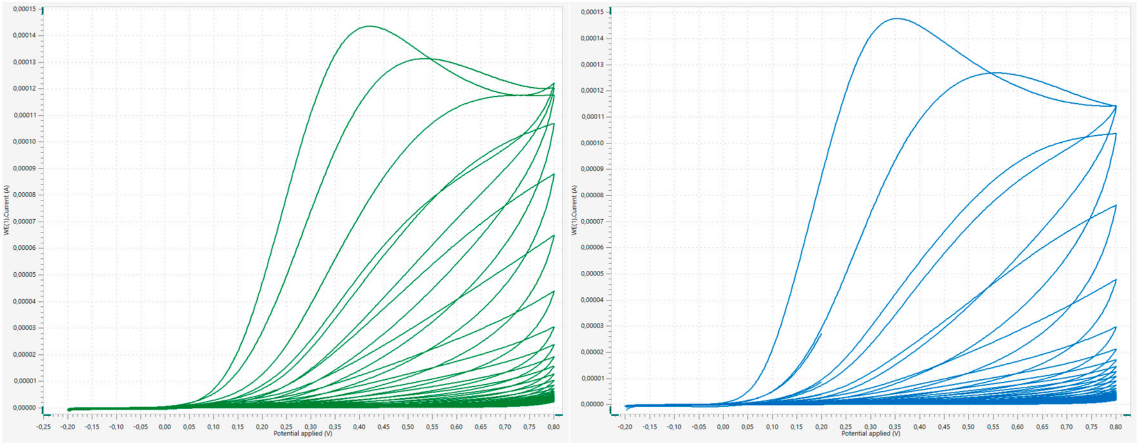

a) **b**)

Figure S1. 40 scans in cyclic voltammetry (CV) for inducing electropolymerization of **a)** DA/LEV (10: 8 mmol L^{-1}) and **b)** DA (10 mmol L^{-1}) on SPCE surface.

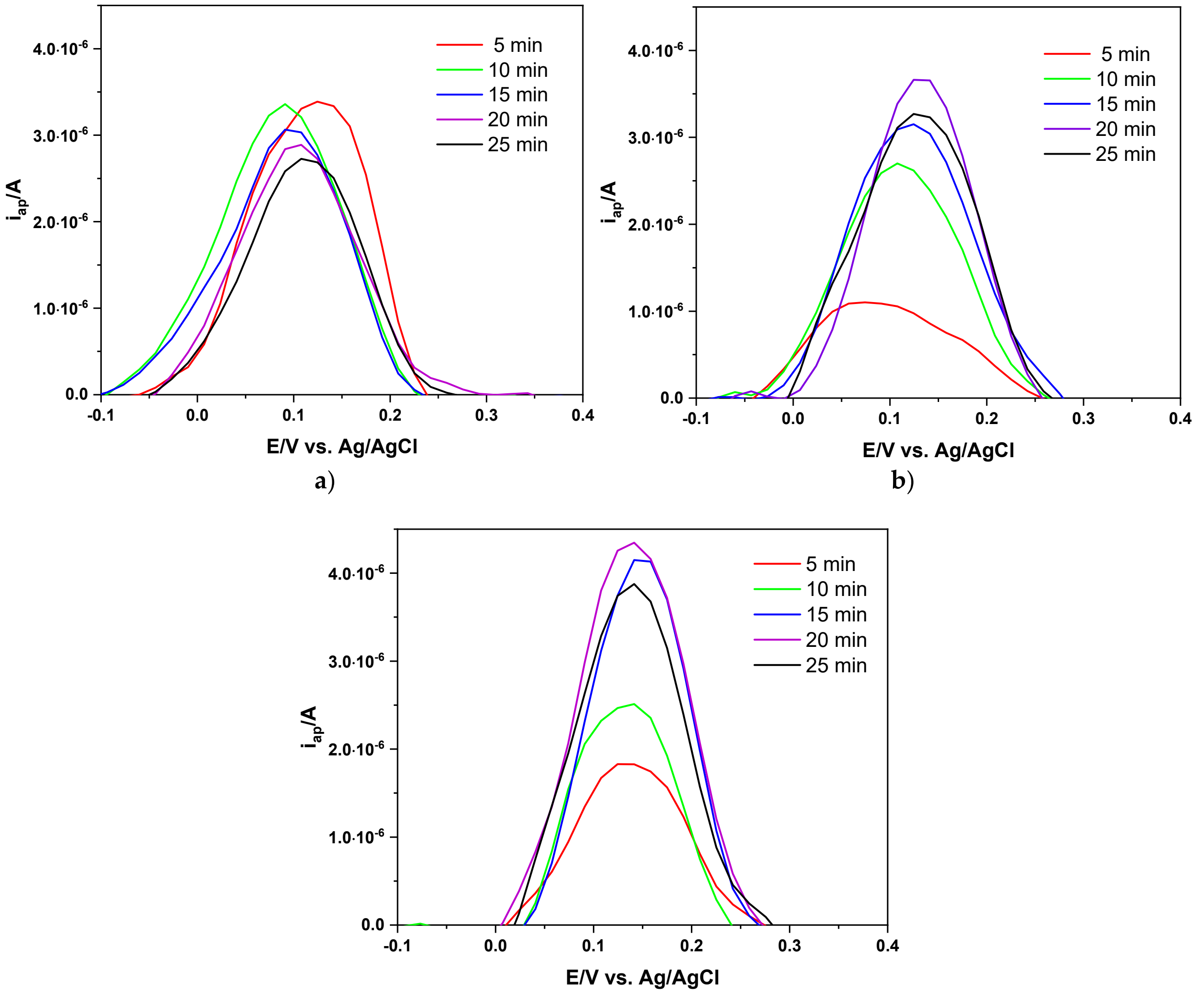

c)

Figure S2. DPV scans of $[Fe(CN)_6]^{4-/3-}$ (5 mmol L^{-1}) in KCl (0.1 mol L^{-1}) on MIP-based SPCE after a washing period between 5 and 25 min in **a)** deionized water, **b)** H_2O/MeOH 1:1 v/v, and **c)** CH_3COOH/ MeOH 1:9 v/v).

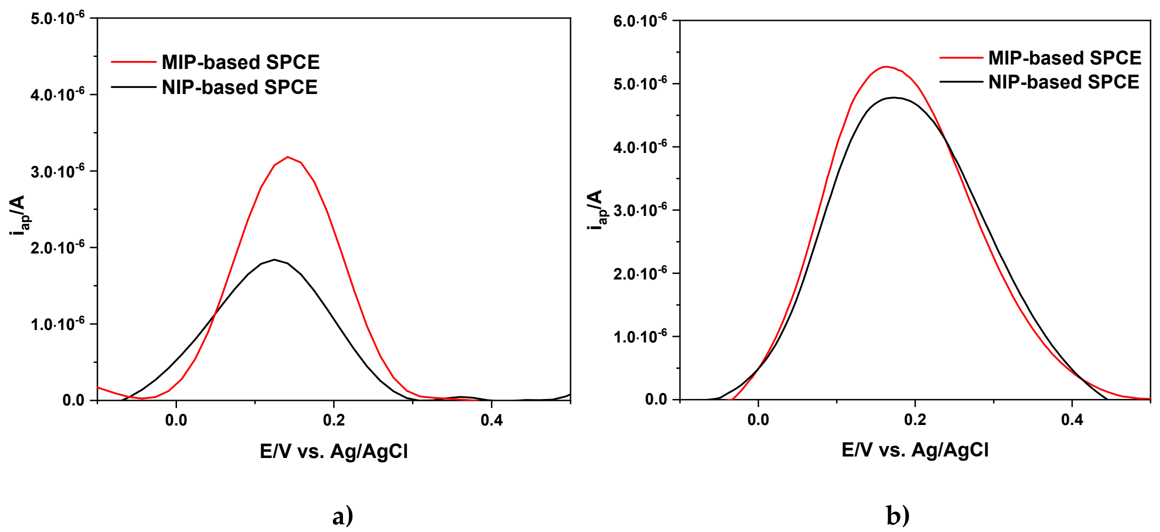

Figure S3. DPV scans of $[Fe(CN)_6]^{4-/3-}$ (5 mmol L^{-1}) in KCl (0.1 mol L^{-1}) on MIP- (red line) and NIP-based SPCE (black line) obtained through **a)** electropolymerization and **b)** drop casting after a period of incubation (15 min) in PB solution containing LEV (0.1 mmol L^{-1}).

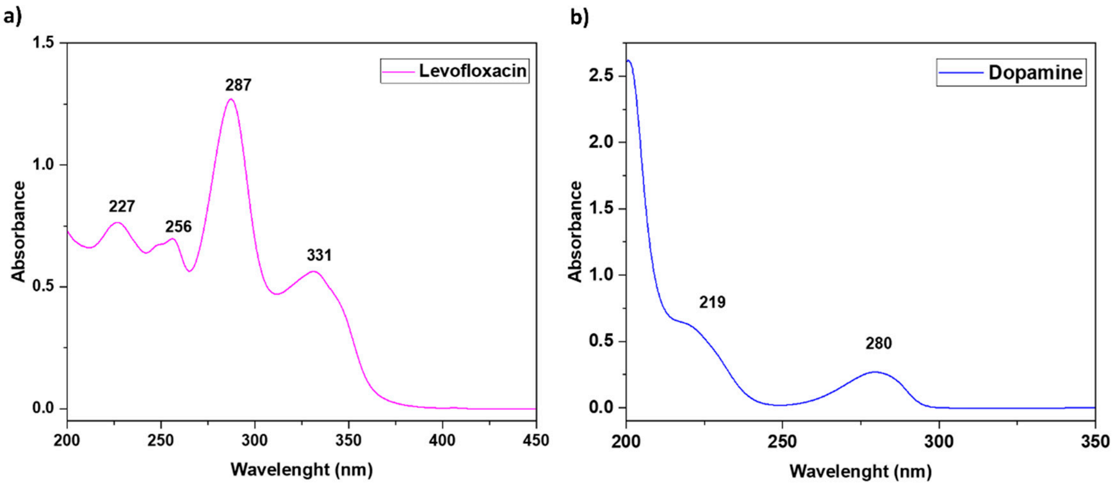

Figure S4. UV-vis spectra of LEV **a)** and DA **b)** recorded in MqW.

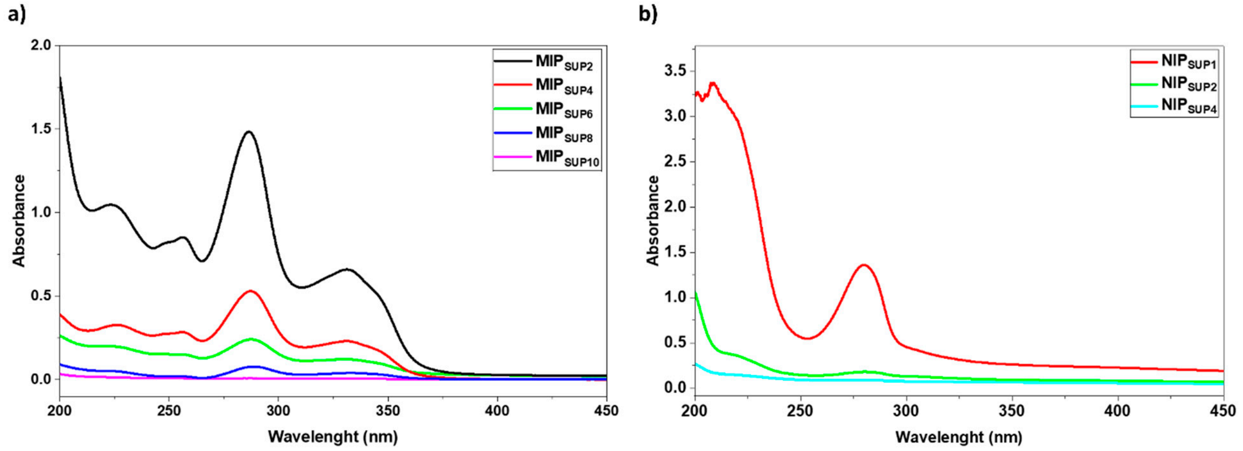

Figure S5. UV-vis spectra of the supernatant after 2, 4, 6, 8 and 10 washing cycles for **a)** MIP and after 1, 2 and 4 washing cycles for NIP **b)**.

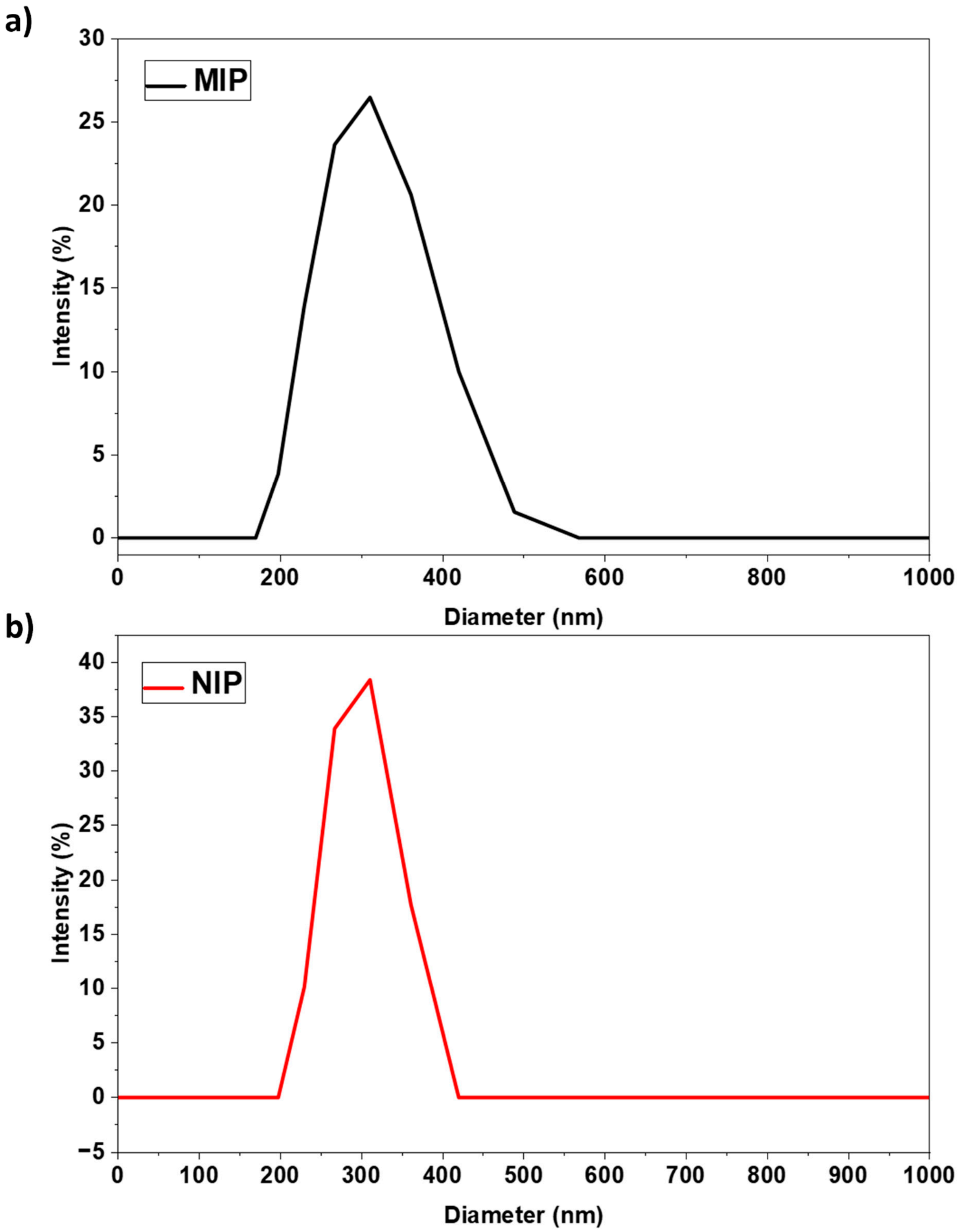

Figure S6. The distribution size of both MIP **a)** and NIP **b)** was obtained by DLS measurements, performed at the same sample concentration of 5 μg mL^{-1}.

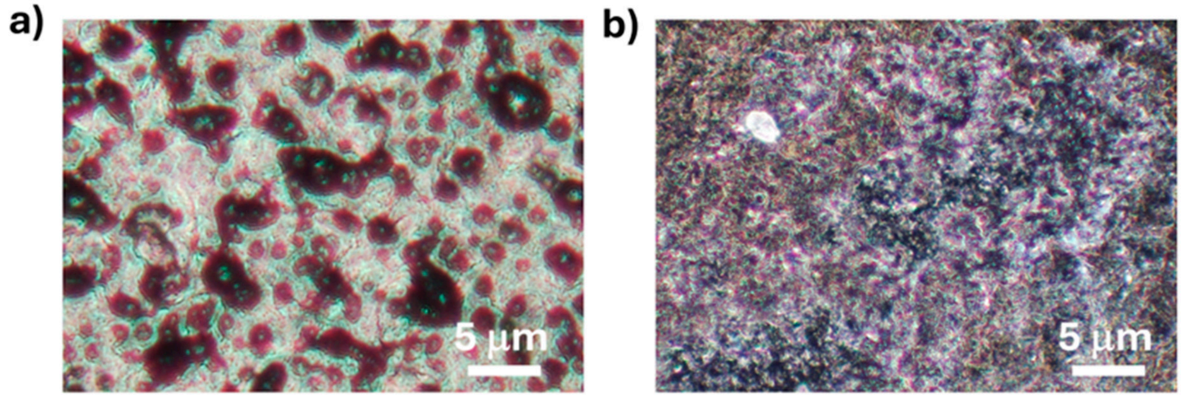

Figure S7. Comparison of the surface morphology of MIP-based SPCEs prepared by **a)** drop casting and **b)** electropolymerization approaches. The images were collected by using Hirox 3D digital microscope.

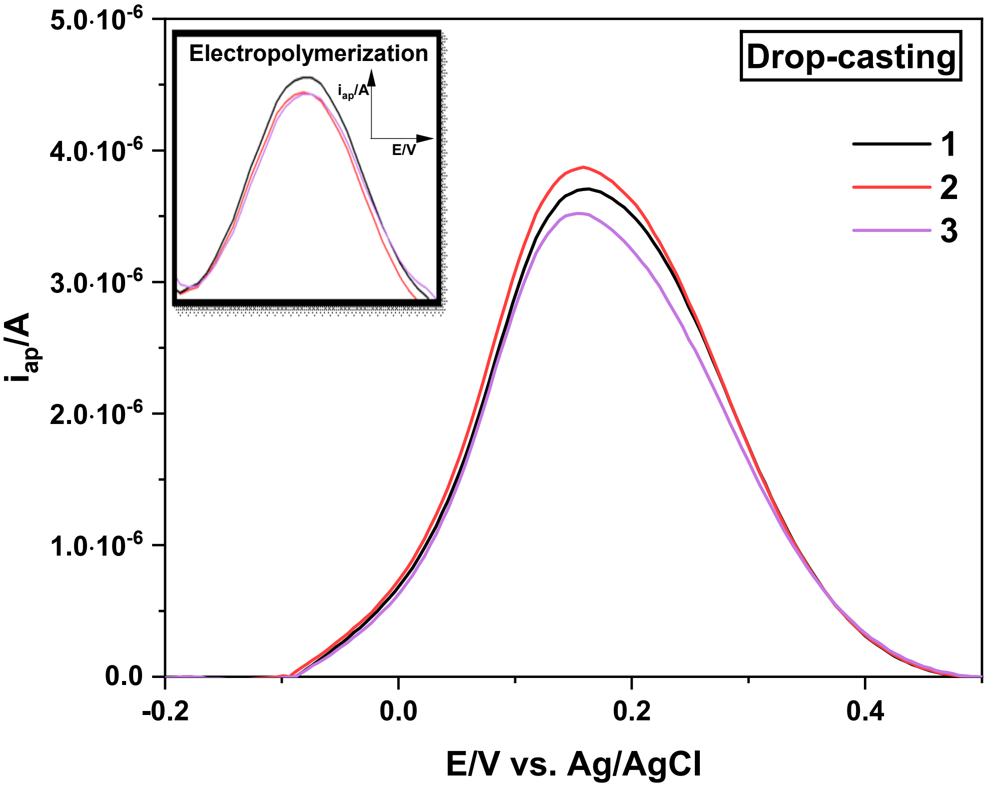

Figure S8. Three DPV scans of $[Fe(CN)_6]^{4-/3-}$ (5 mmol L^{-1}) in KCl (0.1 mol L^{-1}) on MIP- based SPCE, obtained by drop casting, after a period of incubation (15 min) in a PB solution containing LEV (0.26 µmol L^{-1}). The inset shows three DPV scans of $[Fe(CN)_6]^{4-/3-}$ (5 mmol L^{-1}) in KCl (0.1 mol L^{-1}) on MIP- based SPCE, obtained by electropolymerization, after a period of incubation (15 min) in a PB solution containing LEV (0.26 µmol L^{-1}). Each measurement was interspersed with H_2SO_4 (0.1 mol L^{-1}) washes.

Table S1. Potential values and peak currents for $K_3Fe(CN)_6$ (5 mmol L^{-1}) in KCl (0.1mol L^{-1}) obtained on bare SPCE and those obtained by drop-casting of 5 drops (each of 1 μL) of PB solutions containing MIP and NIP (2 mg mL^{-1}), previously synthetized.

	**MIP-based SPCE**				
**Electrode**	**Addition**	**E_{ap}/mV**	**i_{ap}/μA**	**E_{cp}/mV**	**$-i_{cp}$/μA**
**Bare SPCE**	**0**	221	73.1	47	90.3
**MIP-based/SPCE_1**	**1**	325	51.2	-0.07	66.2
**MIP-based/SPCE_2**	**2**	344	49.7	-0.073	64.9
**MIP-based/SPCE_3**	**3**	348	49.3	-0.083	63.5
**MIP-based/SPCE_4**	**4**	372	47.3	-0.089	61.5
**MIP-based/SPCE_5**	**5**	375	45.1	-0.089	59.6
	**NIP-based SPCE**				
**Electrode**	**Addition**	**E_{ap}/mV**	**i_{ap}/μA**	**E_{cp}/mV**	**$-i_{cp}$/μA**
**Bare SPCE**	**0**	219	63.4	26	84.6
**NIP-based/SPCE_1**	**1**	364	47.3	-156	64.9
**NIP-based/SPCE_2**	**2**	442	47.2	-218	60.6
**NIP-based/SPCE_3**	**3**	467	45.8	-226	58.2
**NIP-based/SPCE_4**	**4**	487	43.2	-232	54.5
**NIP-based/SPCE_5**	**5**	508	42.9	-242	52.9

Table S2. Linear concentration range and LOD values reported in the literature for LEV, by MIP-based electrochemical sensing, over the last 12 years.

Electrode	Technique	Linear concentration range ($\mu mol\ L^{-1}$)	LOD value ($\mu mol\ L^{-1}$)	Ref.
MIP/G-Au modified glassy carbon electrode (GCE)[(i)]	Differential Pulse Voltammetry (DPV)	1-100	0.53	[1]
MIP/Au-NPs pencil graphite electrode (PGE)	DPV	1-100	0.462	[2]
MIP/Au-fMWCNT-modified graphite-epoxy composite (GEC)[(ii)]	DPV	2-300	~1	[3]
MIP/di methyl adepate (DMA) ion selective membrane electrode	Potentiometry	50-10000	200	[4]
MIP/acetophenone (AOPH) ion selective membrane electrode	Potentiometry	50-10000	400	[4]
MIP-based ion selective electrode using polyvinyl chloride (PVC)	Potentiometry	10-10000	7.41	[5]

[(i)] Graphene-Au nanoparticles (G-AuNPs).
[(ii)] Au-nanoparticle-decorated multiwall carbon naotubes.

1. Wang, F.; Zhu, L.; Zhang, J., Electrochemical sensor for levofloxacin based on molecularly imprinted polypyrrole–graphene–gold nanoparticles modified electrode. *Sens. Actuators B Chem.* **2014,** 192, 642-647.
2. El Azab, N. F.; Mahmoud, A. M.; Trabik, Y. A., Point-of-care diagnostics for therapeutic monitoring of levofloxacin in human plasma utilizing electrochemical sensor mussel-inspired molecularly imprinted copolymer. *J. Electroanal.l Chem.* **2022,** 918, 116504.
3. Wang, M.; Cetó, X.; del Valle, M., A Sensor Array Based on Molecularly Imprinted Polymers and Machine Learning for the Analysis of Fluoroquinolone Antibiotics. *ACS Sens.* **2022,** *7*, 3318-3325.
4. Abdullah, S.; Al-Bayati, Y. K., Synthesis of new levofloxacin selective membrane sensor based on molecularly imprinted polymers. *IJMRCP* **2021,** 13, 95-107.
5. Mahmoud, A. M.; Saleh, H.; Fawzy, M. G.; Reda, A.; Bahgat, E. A., Design of Potentiometric Sensor Based on Molecularly-imprinted Polymer for Direct Detection of Levofloxacin in Plasma for Point-of-Care Applications. *Zagazig J. Pharm. Sci.* **2024,** 33, 38-48.
